# FAS receptor regulates NOTCH activity through ERK-JAG1 axis activation and controls oral cancer stemness ability and pulmonary metastasis

**DOI:** 10.1038/s41420-022-00899-5

**Published:** 2022-03-05

**Authors:** Li-Jie Li, Peter Mu-Hsin Chang, Chien-Hsiu Li, Yu-Chan Chang, Tsung-Ching Lai, Chia-Yi Su, Chi-Long Chen, Wei-Min Chang, Michael Hsiao, Sheng-Wei Feng

**Affiliations:** 1 Ph.D. Program of School of Dentistry, College of Oral Medicine, Taipei Medical University, Taipei, Taiwan; 2 Genomics Research Center, Academia Sinica, Taipei, Taiwan; 3 Department of Oncology, Taipei Veterans General Hospital, Taipei, Taiwan; 4 Faculty of Medicine, College of Medicine, National Yang Ming Chiao Tung University, Taipei, Taiwan; 5 Institute of Biopharmaceutical Sciences, National Yang Ming Chiao Tung University, Taipei, Taiwan; 6 Department of Biomedical Imaging and Radiological Sciences, National Yang Ming Chiao Tung University, Taipei, Taiwan; 7 Division of Pulmonary Medicine, Department of Internal Medicine, Wan Fang Hospital, Taipei Medical University, Taipei, Taiwan; 8 Department of Biomedical Engineering, Johns Hopkins University, Baltimore, MD USA; 9 Department of Pathology, College of Medicine, Taipei Medical University, Taipei, Taiwan; 10 Department of Pathology, Taipei Medical University Hospital, Taipei Medical University, Taipei, Taiwan; 11 School of Oral Hygiene, College of Oral Medicine, Taipei Medical University, Taipei, Taiwan; 12 Department of Biochemistry, Kaohsiung Medical University, Kaohsiung, Taiwan; 13 Ph.D. Program of Translational Medicine, Taipei Medical University, Taipei, Taiwan; 14 School of Dentistry, College of Oral Medicine, Taipei Medical University, Taipei, Taiwan; 15 Division of Prosthodontics, Department of Dentistry, Taipei Medical University Hospital, Taipei, Taiwan

**Keywords:** Oral cancer, Metastasis, Cancer stem cells

## Abstract

Pulmonary metastasis occurring via the colonization of circulating cancer stem cells is a major cause of oral squamous cell carcinoma (OSCC)-related death. Thus, understanding the mechanism of OSCC pulmonary metastasis may provide a new opportunity for OSCC treatment. FAS, a well-known apoptosis-inducing death receptor, has multiple nonapoptotic, protumorigenic functions. Previously, we found that SAS OSCC cells with FAS receptor knockout did not affect orthotopic tumor growth or cervical lymph node metastasis. However, FAS knockout cells could not colonize in distant organs to form metastases upon intravenous injection, which hinted at the cancer stemness function of the FAS receptor. Immunohistochemistry staining indicated that the FAS receptor serves as a poor prognosis marker in OSCC patients. FAS knockout inhibited in vitro cancer spheroid formation, migration and invasion, and prevented mesenchymal transition in OSCC cells and inhibited OSCC pulmonary metastasis in vivo. To determine the regulatory mechanism by which the FAS receptor exerts its oncogenic function, we utilized cDNA microarrays and phosphoprotein arrays to discover key candidate genes and signaling pathway regulators. JAG1 expression and NOTCH pathway activation were controlled by the FAS receptor through ERK phosphorylation. Both JAG1 and NOTCH1 silencing decreased in vitro cancer spheroid formation. In OSCC cells, FAS ligand or JAG1 protein treatment increased NOTCH pathway activity, which could be abolished by FAS receptor knockout. In FAS knockout cells, restoring the NOTCH1 intracellular domain stimulated cancer spheroid formation. Both JAG1 and NOTCH1 silencing decreased in vivo OSCC growth. In conclusion, we found a novel FAS-ERK-JAG1-NOTCH1 axis that may contribute to OSCC stemness and pulmonary metastasis.

## Introduction

Oral squamous carcinoma (OSCC) is the fifth most common cancer in Taiwanese males and the eighth-most common cancer in the United States. Although cancer treatment strategies have significantly improved in recent decades, OSCC still causes nearly 3000 deaths in Taiwan and 10,000 deaths in the United States each year [1]. OSCC could be considered a locoregional disease; however, OSCC distant metastasis is a major determinant of treatment management strategies and cancer prognosis [2]. OSCC-related death is closely associated with local recurrence or distant metastasis. In the process of metastasis, OSCC cells from the primary tumor migrate to cervical lymph nodes [3] and other organs, such as the lung, bone, liver, and mediastinum. Up to 80% of distant metastases occur in the lung [4, 5]. Thus, understanding the mechanism of OSCC lung metastasis is important in OSCC prognosis evaluation and proving potential therapeutic targets [6, 7]. Although the reason of OSCC recurrence is still unclear, cancer stem cells (CSCs) have been reported as the most crucial player in these steps, especially distant metastasis [8, 9].

FAS cell surface death receptor (FAS) is a member of the TNF-receptor superfamily and plays a crucial role in programmed cell death, which is triggered by FAS ligand (FASLG) [10]. In cell apoptosis, FAS forms a death-inducing signaling complex (DISC) with FADD (Fas-associated death domain protein) and caspase 8 through the DED domain by receptor’s death domains to promote downstream apoptosis signaling [11, 12]. Tumor cells usually suppress the cell surface expression of the FAS receptors to escape apoptosis, which triggers immune cell infiltration [13]. However, the complete loss of FAS expression is hardly observed in human cancer [14]. FAS has a nonapoptotic role in T cells, thymocytes, fibroblasts, and hepatocytes, as it stimulates their proliferation [15–17]. Additionally, FAS promotes metastatic spread in pancreatic ductal adenocarcinoma [18] and maintains cancer stemness [19, 20]. Therefore, a low baseline level of FAS/FASLG signaling is necessary for the survival of cancer cells [21, 22]. FAS also induces extracellular signal-regulated kinase (ERK) activation in caspase activity-independent events [23]. However, the oncogenic role of FAS tumorigenesis and cancer progression has not been studied in OSCC.

Jagged1 (JAG1) is strongly expressed in the highly proliferative types of oral epithelial strata, such as the basal stratum and stratum spinosum [24]. JAG1 is one of the NOTCH ligands that promote serial proteases such as a disintegrin and metalloprotease domain 10 (ADAM10) and γ-secretase complex activation and leads to NOTCH intracellular domain (NICD) release and transcriptional activation [25]. In tumorigenesis and cancer progression, JAG1 is involved in CSC functions, immune regulation, cancer proliferation, angiogenesis, epithelial-to-mesenchymal transition (EMT), and metastasis [26]. Moreover, JAG1 plays a role in the tumor microenvironment. Tumors surrounding endothelial cells [27], osteoblasts [28], and myeloid-derived suppressor cells [29] have high JAG1 activity. Recently, JAG1 has been identified as a therapeutic target in breast cancer [30].

Previously, we revealed that FAS knockout in OSCC cells did not affect orthotopic tumor growth or cervical lymph node metastasis [31]. Here, we found that FAS knockout suppresses OSCC stemness ability by silencing JAG1-NOTCH1 pathway activity regulated by ERK phosphorylation. Importantly, this process is closely associated with the mechanism of OSCC pulmonary metastasis and the poor prognosis of OSCC patients.

## Results

### Knockout FAS receptor suppresses oral squamous cell carcinoma (OSCC) progression

**Fig. 1:**
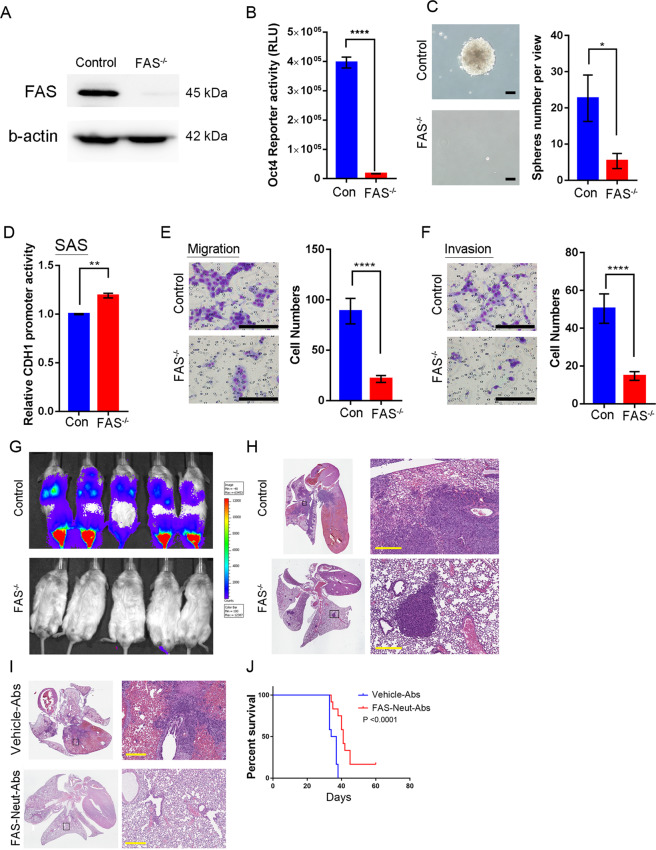
The FAS receptor regulates in vitro OSCC stemness and progression and in vivo lung colony formation. **A** Western blot analysis of FAS and β-actin protein expression in control and FAS^−/−^ SAS cells. **B** OCT4 reporter activity between control and FAS^−/−^ SAS cells. **C** CSC sphere formation assay between control and FAS^−/−^ SAS cells. Scale bar: 100 μm. **D** CDH1 reporter activity between control and FAS^−/−^ SAS cells. **E**, **F** Cellular migration (**E**) and invasion (**F**) ability of SAS FAS^−/−^ cells. Scale bar: 100 μm. **G** IVIS images of SAS-Luc control and FAS^−/−^-Luc cells (*n* = 5/group)**. H** Lung H&E staining of SAS-Luc control and FAS^−/−^ cells. H&E staining reveals high pulmonary hemorrhage and diffuse cancer growth. Scale bar: 300 μm. **I** H&E staining of parental SAS lung colony formation assay with or without FAS neutralizing antibody (FAS-Neut-Ab) pretreatment. Scale bar: 300 μm. **J** The mouse survival curve of parental SAS cells with or without FAS-Neut-Ab pretreatment.

To examine the oncogenic function of the FAS receptor, we performed the CRISPR/Cas9 knockout system to disturb mature FAS protein expression. SAS cells have not only higher-level FAS receptor expression [31] but also high in vitro stemness formation ability [32] and in vivo malignancy in Nod-SCID mice [3]. To increase the chance of immature FAS protein production, we designed the FAS receptor sgRNA to target the second intron-exon junction, specifically the region between cystine 63 and glycine 66 of the FAS receptor primary sequence. FAS receptor knockout (FAS^−/−^) deleted both alleles and induced the early termination of FAS transcription. FAS receptor knockout cells revealed no FAS receptor expression (Fig. 1A). According to our previous study [31], SAS cells have high FAS receptor expression but low FASLG expression, while Cal-27 cells have low FAS expression but high FASLG expression. We examined the stemness ability of FAS^−/−^ SAS cells and found that FAS depletion also decreased OCT4 reporter activity (Fig. 1B) and prevented cancer spheroid formation (Fig. 1C). Compared to control SAS cells, FAS^−/−^ cells increased CDH1 reporter activity and significantly decreased cell migration and invasion (Fig. 1D–F). In contrast to SAS cells, Cal-27 FASLG^−/−^ cells showed downregulated CDH1 promoter activity and increased cell migration and invasion in Boyden chamber assays (Supplementary Fig. 1A–C). These results indicated that both FAS and FASLG may affect OSCC CSC abilities and epithelial-mesenchymal transition (EMT) through transcriptional suppression of the CDH1 promoter.

### FAS receptor regulates in vivo OSCC pulmonary colonization ability

Previously, we found that FAS receptor knockout did not inhibit orthotropic growth or cervical lymph node metastasis [31]. However, when we measured the lung colony formation ability in a tail vein injection model mimicking OSCC distant metastasis, surprisingly, we found that the injection of FAS^−/−^ SAS dramatically reduced the number of lung colonies and cancer cell colonies in the intrafemoral region compared with that with the injection of control SAS cells (Fig. 1G). Highly malignant SAS cells induced aberrant pulmonary hemorrhage and led to diffuse cancer colonies under hematoxylin and eosin (H&E) staining (Fig. 1H upper), but nonetheless, FAS receptor knockout revealed only a few cancer colonies and limited margins compared with that in the control group (Fig. 1H lower). Furthermore, we found that FAS receptor expression is a poor prognostic marker in terms of short-term HNSCC survival according to The Cancer Genome Atlas (TCGA) database [33], and we also found that it may correlate with the metastasis-free period in clinical OSCC patients (Supplementary Figs. 2–4) [34]. Blockade of the FASLG-FAS interaction by FAS neutralizing antibody pretreatment suppressed OSCC lung colony formation and malignant pulmonary hemorrhage (Fig. 1I) and extended mouse survival time (Fig. 1J). Taken together, these results suggest that the FAS receptor controls OSCC pulmonary metastasis by increasing stemness potential, and extraversion ability.

### FAS is a poor prognosis marker in OSCC patients

**Fig. 2:**
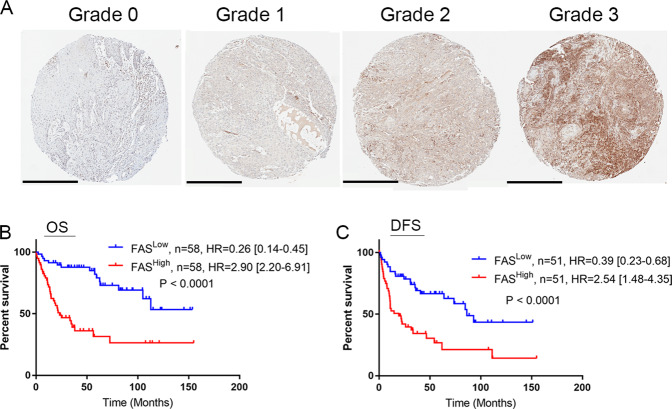
FAS is a poor prognostic marker in OSCC patients. **A** Representative IHC images of FAS expression in OSCC patients. Grade 0 indicates the weakest FAS expression, and grade 3 indicates the strongest FAS expression. Scale bar: 400 μm **B**, **C** Kaplan–Meier plots of the OS (**B**) and DFS (**C**) of OSCC patients.

To clarify the clinical relevance of FAS in OSCC patients, we examined FAS protein expression in Taiwanese OSCC patients’ specimens. Typical FAS staining patterns in OSCC tumors meeting the defined scoring criteria are shown in Fig. 2A. Our results revealed that patients with high FAS expression (score of 2 or 3) had significantly shorter overall and disease-free survival (DFS) times than those with low FAS expression (score of 0 or 1, *p* < 0.0001). The hazard ratio of overall survival (OS) was 2.9 [95% confidence interval (95% CI) = 2.20–6.91] in patients with high FAS expression and 0.26 [95% CI = 0.14–0.45] in patients with low expression (Fig. 2B). In terms of DFS, the hazard ratio was 2.54 [95% CI = 1.48–4.35] in patients with high FAS expression and 0.39 [95% CI = 0.23–0.68] in patients with low expression (Fig. 2C). Furthermore, FAS expression was correlated with clinical T stage, N stage, and differentiation status in OSCC patients (Supplementary Table 2). Overall, these results demonstrated that FAS upregulation could serve as a poor prognostic marker in OSCC patients.

### FAS promotes the JAG1-NOTCH1 signaling pathway in OSCC cells

**Fig. 3:**
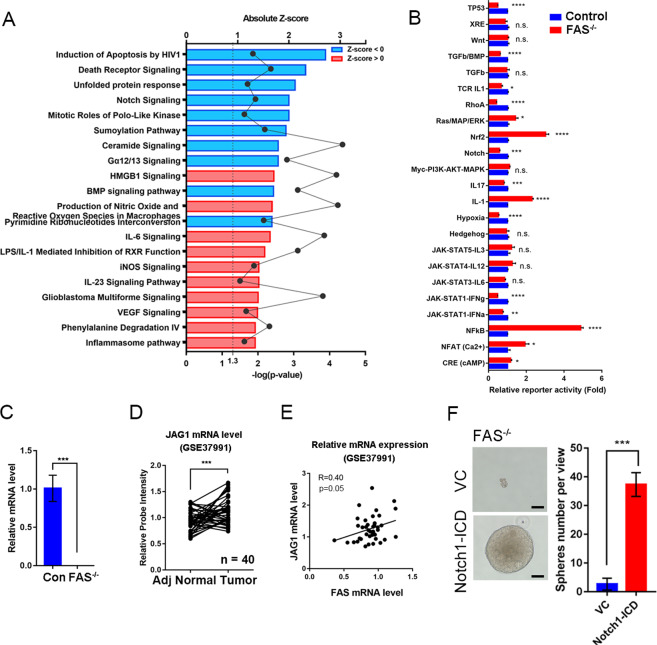
FAS regulates OSCC stemness through NOTCH signaling pathways. **A** Global mRNA expression in FAS^−/−^ SAS cells analyzed by an Affymetrix U133 cDNA microarray. The significantly differentially expressed genes (fold change = 2) were further analyzed by IPA upstream regulator in *z*-score to reveal the differential signaling pathways in FAS^−/−^ cells. **B** Promoter reporter assays of cancer-related signaling pathways between control and FAS^−/−^ SAS cells. **C** *JAG1* mRNA expression level in control and FAS^−/−^ SAS cells. **D** *JAG1* mRNA expression in OSCC patients **E** Pearson correlation analysis of *FAS* and *JAG1* mRNA expression in OSCC patients. **F** The CSC sphere formation assay in FAS^−/−^ SAS cells with or without restoration of the NOTCH1-ICD. Scale bar: 100 μm.

To determine the mechanism by which FAS stimulates OSCC stemness and extravasation and impacts OSCC progression, we performed a cDNA microarray analysis of control and FAS^−/−^ cells by ingenuity pathway analysis (IPA). The OSCC FAS-relative gene signature was defined by screening genes with a fold change ≥2-fold in FAS^−/−^ cells versus control cells. There were 3065 probes that matched this limitation (Supplementary Table 3). The bioinformatics results showed that FAS knockout suppressed several signaling and metabolic pathways (Fig. 3A and Supplementary Table 4). We used the *z*-score analysis by downstream gene expressions profiling. We found the top two ranking signatures, “Induction of Apoptosis by HIV1” and “Death Receptor Signaling” were highly relative to FAS cell death function which convinced us of the FAS knockout signatures. The “Unfolding Protein Response” is an endoplasmic reticulum (ER) function alternation that causes accumulation of unfolded or misfolded proteins which is a global cell impact which could not predict a single factor contribution [18]. After combining with the transcriptional activity alternations by common oncogenic pathway analysis in FAS knockout cells, we found “TP53”, “TGFβ/BMP”, “TCR-IL1”, “RhoA”, NOTCH”, “IL17”, “hypoxia”, “JAK-STAT1-IFNα”, and “JAK-STAT1-IFNγ” were silenced in FAS knockout cells (Fig. 3B). Interestingly, the NOTCH ligand *JAG1* was also downregulated according to the cDNA microarray results (Supplementary Table 3). Thus, we validated *JAG1* expression by qPCR analysis. *JAG1* mRNA expression was significantly reduced in FAS knockout cells compared with control cells (Fig. 3C). In paired OSCC samples, *JAG1* mRNA levels were upregulated in tumor tissues compared to adjacent normal tissues (Fig. 3D, GSE37991). Moreover, we found a positive correlation between *FAS* and *JAG1* mRNA in Taiwanese OSCC patients (Fig. 3E). When NOTCH signaling is triggered by JAG1-NOTCH1 binding, the NOTCH1 intracellular domain (NOTCH1-ICD) is cleaved by γ-secretase, translocates into the nucleus, and drives NOTCH signaling pathway genes [35]. We restored NOTCH1-ICD in FAS^−/−^ cells and found that NOTCH1-ICD recovered OSCC stemness ability (Fig. 3F). These results indicate that the JAG1-NOTCH1 signaling pathway may be crucial in the mechanism by which FAS regulates cancer stemness in OSCC cells.

**Fig. 4:**
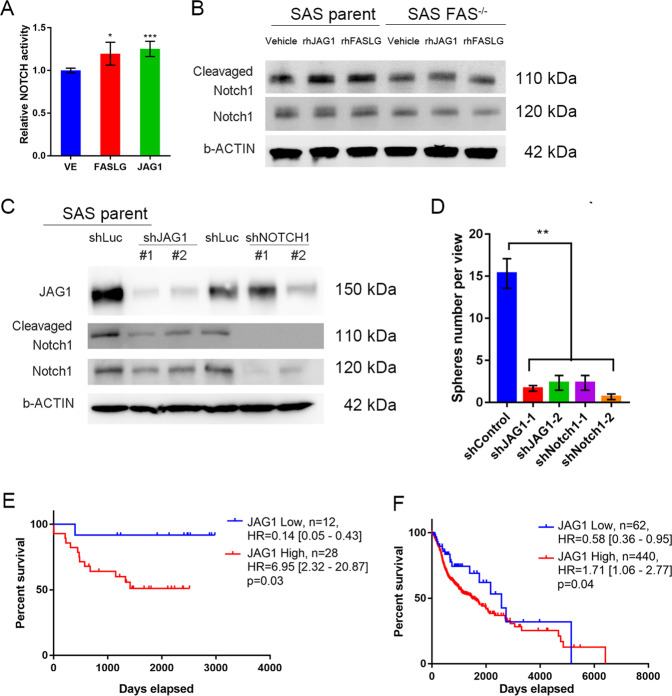
rhJAG1 or rhFASLG treatment controls NOTCH signaling activation. **A** rhJAG1 (0.25 ng/mL) or rhFASLG (0.25 ng/mL) treatment promotes NOTCH1 signaling activation. **B** NOTCH signaling pathway activation analysis by cleavage NOTCH1 expression under rhJAG1 or rhFASLG treatment. **C** Knockdown of JAG1 or NOTCH1 by shRNA suppresses NOTCH1-ICD cleavage. **D** The CSC sphere formation assay in JAG1- or NOTCH1-silenced SAS cells. **E**, **F** JAG1 Kaplan–Meier plots of OS for the Taiwanese OSCC cohort (**E**, GSE37991) and the TCGA-HNSC cohort (**F**).

**Fig. 5:**
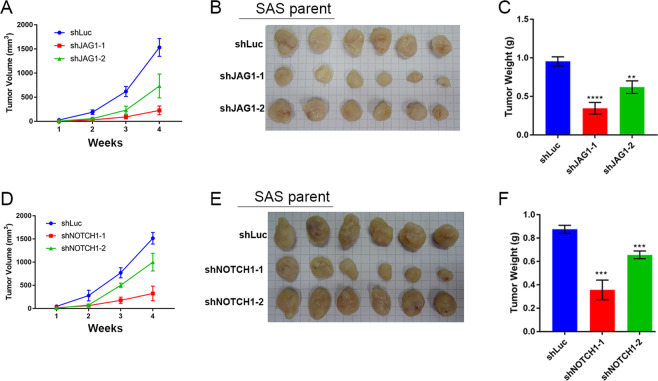
Silencing of JAG1 or NOTCH1 suppresses in vivo OSCC growth. **A** In vivo tumor burden of mice injected with JAG1-silenced SAS cells. **B**, **C** Representative tumor picture (**B**) and tumor weight (**C**) for **A**. **D** In vivo tumor burden of NOTCH1-silenced SAS cells. **E**, **F** Representative tumor picture (**E**) and tumor weight (**F**) for **D**. (*n* = 6/group).

We further dissected the molecular regulation mechanism in OSCC, and we found that recombinant (rh) FASLG promoted NOTCH1 transcription activity, which is similar to the effects of rhJAG1 protein (Fig. 4A). Both FASLG and JAG1 protein treatments activated NOTCH1 signaling via the induction of NOTCH1-ICD protein expression in SAS parental cells. However, NOTCH signaling activation was abolished in FAS knockout (FAS^−/−^) cells (Fig. 4B). We further silenced JAG1 or NOTCH1 expression with shRNA and found cleavage of the NOTCH1-ICD was decreased in JAG1- and NOTCH1-silenced cell lines (Fig. 4C). Moreover, the in vitro OSCC stemness ability was inhibited by JAG1 or NOTCH1 depletion (Fig. 4D). In combination with Fig. 3F and Fig. 4D, we could establish the causal relationship between FAS and NOTCH signaling in OSCC stemness. Interestingly, we found that high JAG1 expression was associated with unfavorable prognostic outcomes in both the Taiwanese cohort (Fig. 4E, GSE37991) and the TCGA head and neck cancer (TCGA-HNSC) cohort (Fig. 4F). Furthermore, we found that JAG1 and NOTCH1 silencing decreased the in vivo OSCC tumor burden and tumor weight, respectively (Fig. 5A–F). In summary, we found that the JAG1 and NOTCH signaling pathways may be key modulators by which FAS maintains OSCC stemness and pulmonary metastasis.

### FAS affects cellular kinase activity in OSCC

**Fig. 6:**
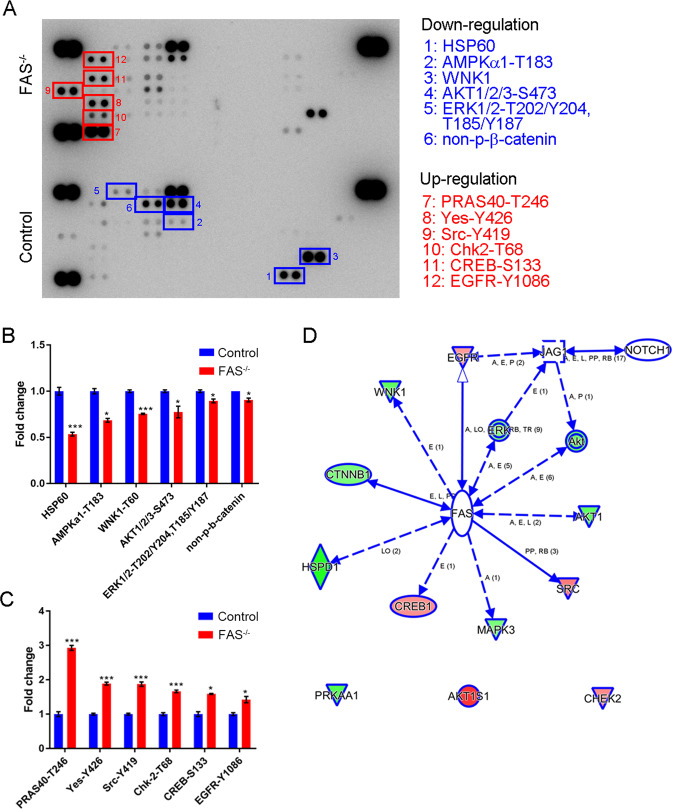
FAS regulates cell survival kinase activation. **A** Representative phosphoprotein array images of control and FAS^−/−^ SAS cells. **B**, **C** The plots of significantly downregulated (**B**) and upregulated kinases and proteins (**C**) from **A**. **D** IPA model showing how FAS regulates JAG1 expression.

**Fig. 7:**
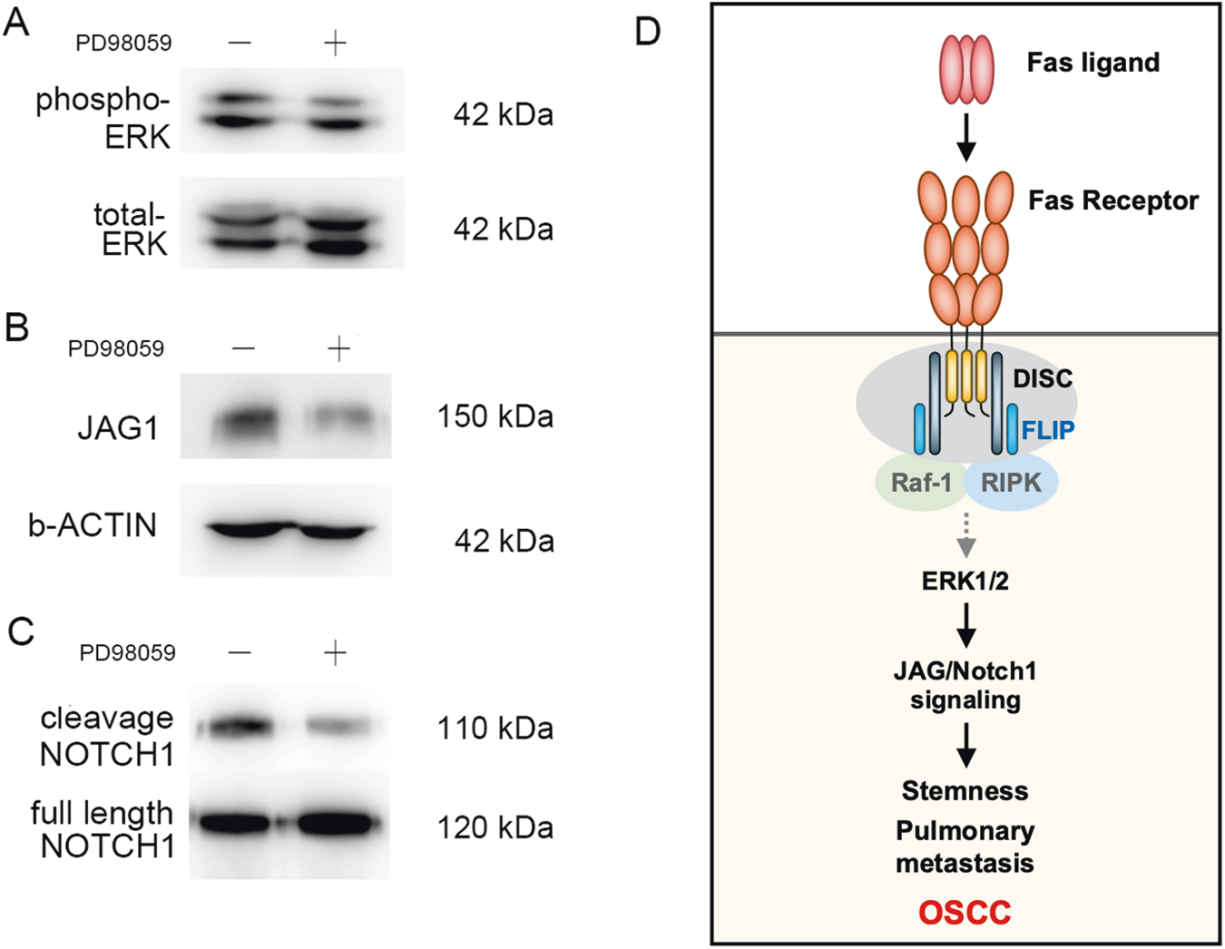
The FAS receptor regulates JAG1 expression in OSCC through ERK activation. **A–C** ERK inhibitor (PD98059 10 μM) treatment inhibited ERK phosphorylation (**A**), JAG1 expression (**B**), and NOTCH1 cleavage (**C**). **D** Hypothetical model showing how FAS controls OSCC distant pulmonary metastasis via the activation of ERK-JAG1- NOTCH1.

Furthermore, we used a commercial human phosphokinase array to study the mechanisms by which FAS regulates OSCC stemness and metastasis (Fig. 6A). Unexpectedly, FAS knockout suppressed the phosphorylation of cancer cell survival signaling molecules, such as AKT-S473, ERK1/2-T202/Y204, ERK1/2-T185/Y187, and AMPKα1-T174. Moreover, the protein expression levels of the active form of β-catenin (nonphosphorylation modification), metastasis kinase WNK1 (T60), and heat shock protein chaperonin 60 (HSP60) were downregulated in FAS^−/−^ cells and tumor growth was inhibited, which indicated that FAS receptor knockout is crucial for regulating the expression of survival-related genes in OSCC (Fig. 6B). Conversely, FAS knockout promoted the expression of a tumor suppressor gene, CHEK2, and disrupted the balance of differentiation-related kinases SRC and YES kinase [36] (Fig. 6C). These changes might reduce cancer survival advantages in FAS^−/−^ cells. To identify the regulators in the FAS-JAG1 axis, we used IPA. We found that ERK phosphorylation was necessary for *JAG1* mRNA expression (Fig. 6D). In parental SAS cells, PD98059, an inhibitor of ERK activation, decreased JAG1 and NOTCH1-ICD protein expression (Fig. 7A–C). Moreover, both RIP kinase inhibitor, Necrostatin-1, and selective ERK inhibitor, FR-180204, suppressed ERK activation and JAG1 expression (Supplementary Fig. 5). Recombinant protein treatment of rhJAG1, rhFASLG also stimulated NOTCH-response element transcription reporter expression (Fig. 4A) and NOTCH activation. Overall, we proved that ERK phosphorylation is critical to JAG1 expression and NOTCH signaling pathway activation, which are involved in FAS-mediated regulation of OSCC stemness and pulmonary metastasis.

## Discussion

Here, we demonstrate the oncogenic role of the death receptor FAS in OSCC pulmonary metastasis through ERK-JAG1-NOTCH signaling (Fig. 7D). Cancer cells usually silence apoptosis-related protein expression to prevent programmed cell death triggered by immune cells [37]. In neuronal stem cells, FAS expression promotes stem cell survival and neuronal specification [38]. In glioblastoma, FAS also recruits SRC kinase, YES, and PI3K to promote invasion [39]. Recently, FAS has been reported to have a tumor-promoting function in cancer stemness [20, 40], proliferation [41], and metastasis [16, 42], which is in line with our studies in OSCC. In patient-derived human breast cancer and glioblastoma neurospheres, FAS increases cancer stemness [20, 40, 43]. Unlike breast cancer, we found STAT1 activity was suppressed in the OSCC cells (Fig. 3B). However, the dramatic upregulation of NF-κB activity may be due to the c-FLIP released by FAS knockout and disassembly DISC. Free N-terminal FLIP fragment can interact with TRAF1/2 induce NF-κB activation [44]. Recently, FADD upregulation or S194 phosphorylation are important prognostic biomarkers in multiple cancer progression, especially in OSCC [45–47]. Moreover, constitutively phosphoryl-mimicking mutation of FADD also enhances Notch-1 signaling in muscle regeneration through promoting ERK phosphorylation is consistent with our finding in oral cancer cells [48]. Recruitment of FLIP and FADD are important mediators in nonapoptotic cancer-promoting functions [49]. Inhibition of FAS signaling by APG101 prevents glioblastoma invasion, increases radiosensitivity in vitro [50], and increases glioblastoma patients’ responses to irradiation [51]. This finding suggests that the FAS neutralizing antibody Kp7-6 or the FAS/FASLG antagonist APG101 may be beneficial for preventing OSCC distant metastasis mediated by FAS or its downstream signaling components. Oncoimmune therapy targeting PD-L1 is a new therapeutic niche in patients [52, 53], combining APG101 and immune checkpoint inhibitors may help OSCC immunotherapy.

In our microarray analysis, there were several signaling pathways affected by FAS knockout but not linked to FAS. FAS knockout suppresses both pyrimidine ribonucleotide interconversion and de novo biosynthesis genes, and these effects have not been reported in the literature. Purine and pyrimidine antimetabolites are common chemotherapy agents in cancer therapy [54]. It is worth studying the regulatory mechanism by which FAS regulates pyrimidine ribonucleotide metabolism in OSCC cells, which may provide an opportunity for treating cancers with aberrant metabolism.

To our knowledge, no studies have found signaling crosstalk between the FAS receptor and NOTCH signaling. Our study is the first to prove that the intrinsic FAS receptor regulates ERK phosphorylation and stimulates JAG1 expression. JAG1 serves as a NOTCH1 ligand and maintains NOTCH activity in OSCC cells. In OSCC, the role of NOTCH1 in OSCC tumorigenesis and progression is controversial. NOTCH1 loss-of-function promotes the OSCC carcinogenesis process in mice [55]. However, upregulation of JAG1, NOTCH1, and downstream targets, such as HES1/HEY1, is found in many OSCC patients [56]. The NOTCH signaling pathway is also related to EMT and stemness [57, 58], which is consistent with our results showing that NOTCH governs FAS-mediated oncogenic functions in OSCC. Recently, an anti-NOTCH antibody has been used in the preclinical treatment of OSCC CSCs [59, 60]. Combination with FAS neutralizing antibodies or antagonists may further enhance the anti-CSC function in OSCC.

In conclusion, FAS protein promotes OSCC stemness, migration, invasion, pulmonary and metastasis and affects patient survival. FAS triggers ERK activation and increased the transcriptional activity of JAG1/NOTCH signaling components, suggesting that FAS serves as a novel transcriptional activator of the NOTCH signaling pathway and that apoptosis resistance in OSCC may allow residual cancer cells to remain, causing treatment failure and recurrence. Aberrant intracellular expression of FAS in OSCC highlights FAS as a potential new prognostic biomarker.

## Materials and methods

### Chemical and vector information

All chemical reagents, kits, antibody sources, and primer sequences are listed in Supplementary Table 1.

### Cell culture and CRISPR knockout FAS cells

Cell culture conditions are described in Supplementary Methods. FAS CRISPR knockout (FAS^−/−^) cells were established in our previous study [31] and in Supplementary Methods.

### Microarray analysis

FAS downstream genes and regulators in OSCC were determined by Affymetrix U133 microarray assay and followed the previous analysis approach [61]. The microarray data were uploaded to the National Center for Biotechnology Information Gene Expression Omnibus (GEO, NCBI, GSE147052). The expression of specific genes was validated by EvaGreen-based qPCR assays.

### Western blotting and real-time PCR (RT-qPCR) assay

Western blotting assays and RT-qPCR assays were performed as previously described in refs. [62] and [63], respectively. The details, the antibody dilution conditions, and the primer sequences are described in Supplementary Methods and in Supplementary Table 1.

### Boyden chamber assay

The migration and invasion ability of OSCC cells were measured by Boyden chamber invasion assay (Neuro Probe Inc, Gaithersburg, MD, USA) as previously described in ref. [3] and in Supplementary methods.

### CSC sphere formation assay

The stemness formation assay followed our previous protocol for OSCC [62] and was described in Supplementary Methods.

### In vivo lung colony formation and mouse survival assays

All animal experiments strictly followed the recommendations in the guidelines for the Care and Use of Laboratory Animals of the National Health Research Institutes (Miaoli, Taiwan). The protocol was approved by the Institutional Animal Care and Use Committee of the Genomic Research Center, *Academia Sinica* (Taipei, Taiwan; protocol no.: ASIACUC-15-06-833). Male NOD-SCID gamma mice aged 5–6 weeks were used in this study. In the in vivo lung colony formation assay, 1 × 10^5^ OSCC cells with a luciferase reporter gene were injected into mice through the tail vein. To measure the signal intensity from the luciferase vector, in vivo tumor images were captured by an IVIS imaging system (Caliper Life Sciences, Hopkinton, MA, USA).

### Immunohistochemistry (IHC) staining

IHC staining of tissue microarrays was performed as described in our previous work [3]. OSCC tissue microarrays were obtained from Taipei Medical University Hospital (Taipei, Taiwan) with Institutional Review Board (IRB) approval (TMU-IRB 99049). The histologic type of head and neck cancer was determined according to the WHO classification. The evaluation of tumor size, local invasion, lymph node involvement, distal metastasis, and final disease stage was performed according to the American Joint Committee on Cancer (AJCC) tumor-node-metastasis (TNM) staging system for OSCC [64]. Follow-up was done for up to 100 months.

### Phosphoproteome array

Phosphoproteome profiling was performed with a human phosphokinase antibody array kit (#ARY003B, R&D Systems, Minneapolis, MN, USA) according to the manufacturer’s instructions. The array was analyzed by ImageJ software. The expression levels were normalized to those in the control group (*n* = 2).

### Reporter assays

The details of reporter assays were described in Supplementary Methods. The common oncogenic pathway reporters were purchased from Promega and are listed in Supplementary Table 1.

### Statistical analysis

All statistical analyses were performed using Student’s *t-*test via SPSS (Statistical Package for the Social Sciences) software unless otherwise stated. The figures were created with Prism 7 software (GraphPad Software Inc., La Jolla, CA, USA). The data were presented as the mean ± standard error of the mean (SEM) from three independent experiments. Survival rates were assessed via the Kaplan–Meier method and the log-rank test. Patient follow-up time was censored if the patient was lost to follow-up. The threshold for statistical significance was set at *p* < 0.05 for all of our analyses.

## Supplementary information

Supplementary information

Supplementary Fig. 1.

Supplementary Fig. 2.

Supplementary Fig. 3.

Supplementary Fig. 4.

Supplementary Fig. 5.

Supplementary Table 1.

Supplementary Table 2.

Supplementary Table 3.

Supplementary Table 4.

Western blots data

Attribution form

Authorship change form

## Data Availability

The datasets used and/or analyzed in this study are available from the corresponding author on reasonable request. The microarray raw data were deposited on Gene Expression Omnibus (GSE147052).

## Materials availability

The materials used and/or analyzed in this study are available from the corresponding author on reasonable request. The microarray raw data were deposited on Gene Expression Omnibus (GSE147052).
