# Supplementary material for: FAS receptor regulates NOTCH activity through ERK-JAG1 axis activation and controls oral cancer stemness ability and pulmonary metastasis
Source: Cell Death Discov. 2022 Mar 5;8:101. doi: 10.1038/s41420-022-00899-5 (PMC8898312; doi:10.1038/s41420-022-00899-5)
Supplement: Supplementary file 1 — Supplementary information [file 41420_2022_899_MOESM1_ESM.docx]

**FAS Receptor Regulates NOTCH Activity through ERK-JAG1 Axis Activation and Controls Oral Cancer Stemness Ability and Pulmonary Metastasis**

Li-Jie Li^1,2^, Peter Mu-Hsin Chang^3,4,5^, Chien-Hsiu Li^2^, Yu-Chan Chang^6^, Tsung-Ching Lai^2,7^, Chia-Yi Su^2,8^, Chi-Long Chen^9,10^, Wei-Min Chang^2, 11#^, Michael Hsiao ^2,12,13#^, Sheng-Wei Feng^14,15#^

**Supplementary methods**

**Cell culture and CRISPR knockout FAS cells**

The human OSCC cell line, CAL 27, was obtained from the American Type Culture Collection (ATCC, USA), and SAS were obtained from the Japanese Collection of Research Bioresources Cell Bank (JCRB, Japan). All cell lines were cultured in standard medium according to the manufacturer’s instructions containing 10% fetal bovine serum (FBS; Gibco, USA), 1% L-glutamine (Gibco, USA), and antibiotics (penicillin and streptomycin; Gibco, USA), and maintained in a humidified atmosphere of 5% CO_2_ at 37 °C. FAS CRISPR knockout (FAS^-/-^) cells were established in our previous study [31]. Briefly, SAS cells were infected with the FAS CRISPR knockout pseudovirus, which targeted the GTTTTGTGTAACATACCTGGAGG sequence in the second exon-intron junction, and single-cell colonies were established. The CRISPR knockout cells were by Sanger sequencing of the target genomic DNA region by PCR. Blunt-end ligation was performed with the HE Swift Cloning Kit, and FAS protein expression was assessed.

**Western blotting**

Cell lysates were kept in lysis buffer, and their concentration was determined by BCA assay (Thermo Fisher Scientific, Waltham, MA, USA). Protein lysates (30 μg) were separated by 10% SDS-PAGE and then electrotransferred to 0.45-μM polyvinylidene difluoride (PVDF) membranes (Merck-Millipore, Burlington, MA, USA). After blocking, the membranes were incubated with primary antibodies at 4 °C overnight, followed by the corresponding secondary antibody for 1 h. The expression signals were visualized using the Immobilon Western Chemiluminescent HRP Substrate (Millipore #WBKLS0500) and detected using the Fujifilm LAS4000 luminescent image analysis system (Fujifilm, Tokyo, Japan). The expression was normalized to that of the internal control (β-actin). The antibody dilution conditions are listed in **Supplementary Table 1**.

**Real-time PCR (RT-qPCR) assay**

RT-qPCR assays were performed as previously described [34]. Total RNA was obtained from the TRIzol (Invitrogen, Waltham, MA, USA) extraction method and measured by a Nanodrop spectrophotometer (Thermo Fisher Scientific, Waltham, MA, USA). Reverse transcription-PCR (RT-PCR) was performed using a SuperScript III kit (Invitrogen) according to the manufacturer’s protocol in Gunster P-08A 0.2 ml 8-well PCR strips (Gunster Biotech Co., New Taipei City, Taiwan). The expression levels were detected with OmicsGreen (OmicsBio, Taipei, Taiwan) and normalized to those of GAPDH. The reaction was performed in 0.1-ml qPCR strip tubes or plates with optical caps or film (Gunster Biotech Co). The primer sequences are listed in **Supplementary Table 1.**

**Boyden chamber assay**

The migration and invasion ability of OSCC cells were measured by Boyden chamber invasion assay (Neuro Probe Inc, Gaithersburg, MD, USA) as previously described in [3]. Briefly, PVDF coated with 10 µg/mL fibronectin on the lower side was used in the migration assay, and PVDF coated with 1 mg/mL Matrigel Basement Membrane Matrix (BD Biosciences, Franklin Lakes, NJ, USA) on the upper side was used in the invasion assay. A total of 1.5 x 10^4^ cells were seeded into the upper chamber for 16 h. After fixation, the invaded cells on the membrane were stained with Giemsa stain (Sigma-Aldrich) and then quantified from at least five images obtained through phase-contrast microscopy.

**CSC sphere formation assay**

The stemness formation assay followed our previous protocol for OSCC [33]. Briefly, stable cells (1×10^3^) were incubated with 2 mL sphere formation medium (DMEM with 20 ng/mL EGF, bFGF and 1X B27 supplement) and seeded in ultralow attachment 6-well plates (Corning) for 14 days. Spheroids were captured with an inverted microscope, and at least five images were finally obtained through phase-contrast microscopy to count the number of spheroids. The spheroids were counted only when the cell number was greater than 50 µm.

**Reporter assays**

CDH1 and OCT4 reporters were purchased from Addgene (Watertown, MA, USA) or System Biosciences (Palo Alto, CA, USA) and packaged into pseudoviral particles in 293T cells. Treated cells (5×10^5^) were infected with the CDH1 or OCT4 reporter and empty pseudovirus. After an additional 48 h of incubation, the reporter activity was determined by One-Glo luciferase substrate and measured with a PerkinElmer EnSpire Alpha plate reader (PerkinElmer, Waltham, MA, USA). Other common oncogenic pathway reporters were purchased from Promega and are listed in **Supplementary Table 1**. Briefly, 2×10^5^ control and FAS knockout cells were seeded in 24-well plates and then transfected with 500 ng oncogenic pathway reporters per well with Lipofectamine 2000 on the second day. After 24 h of incubation, the reporter activity was determined as above method. The reporter activities were normalized to those of the empty control expression in different stable cells.

**Supplementary figure legends**

**Supplementary Fig. 1**.

**FASLG controls *in vitro* OSCC migration, invasion, and stemness ability.**

**A.** CDH1 reporter activity between control and FASLG^-/-^ Cal-27 cells. **B** and **C**. Cell migration **(B)** and invasion assay **(C)** of Cal-27 with or without FASLG expression. Scale bar: 100 μm.

**Supplementary Fig. 2**.

FAS receptor expression is correlate disease stage in TCGA-HNSC cohort.

**Supplementary Fig. 3**.

FAS receptor expression is an early poor prognostic marker in TCGA-HNSC cohort.

**Supplementary Fig. 4**.

FAS receptor expression indicates metastasis-free survival time among OSCC patients. The Kaplan-Meier survival curve was analyzed from Survexpres database.

**Supplementary Fig. 5.**

Both ERK inhibitor (ERKi; FR-180204) and RIP kinase inhibitor (RIPKi; Necrostatin-1) downregulated the protein expression of JAG1 in SAS cells. CT, control. Western blotting showing the protein expression of p-ERK, ERK and JAG1 after ERKi and RIPKi treatments for 6 h in SAS cells.

**Supplementary table legends**

**Supplementary Table 1. Reagents, vectors, antibody dilution conditions and primer information**

**Supplementary Table 2. Clinical characteristics of FAS in validation HNSCC cohort**

**Supplementary Table 3. Differential expressed genes of FAS^-/-^ SAS cells**

**Supplementary Table 4. Differential expressed IPA canonical pathways of FAS^-/-^ SAS cells**
