# Supplementary figures and images for: FAS receptor regulates NOTCH activity through ERK-JAG1 axis activation and controls oral cancer stemness ability and pulmonary metastasis
Source: Cell Death Discov. 2022 Mar 5;8:101. doi: 10.1038/s41420-022-00899-5 (PMC8898312; doi:10.1038/s41420-022-00899-5)

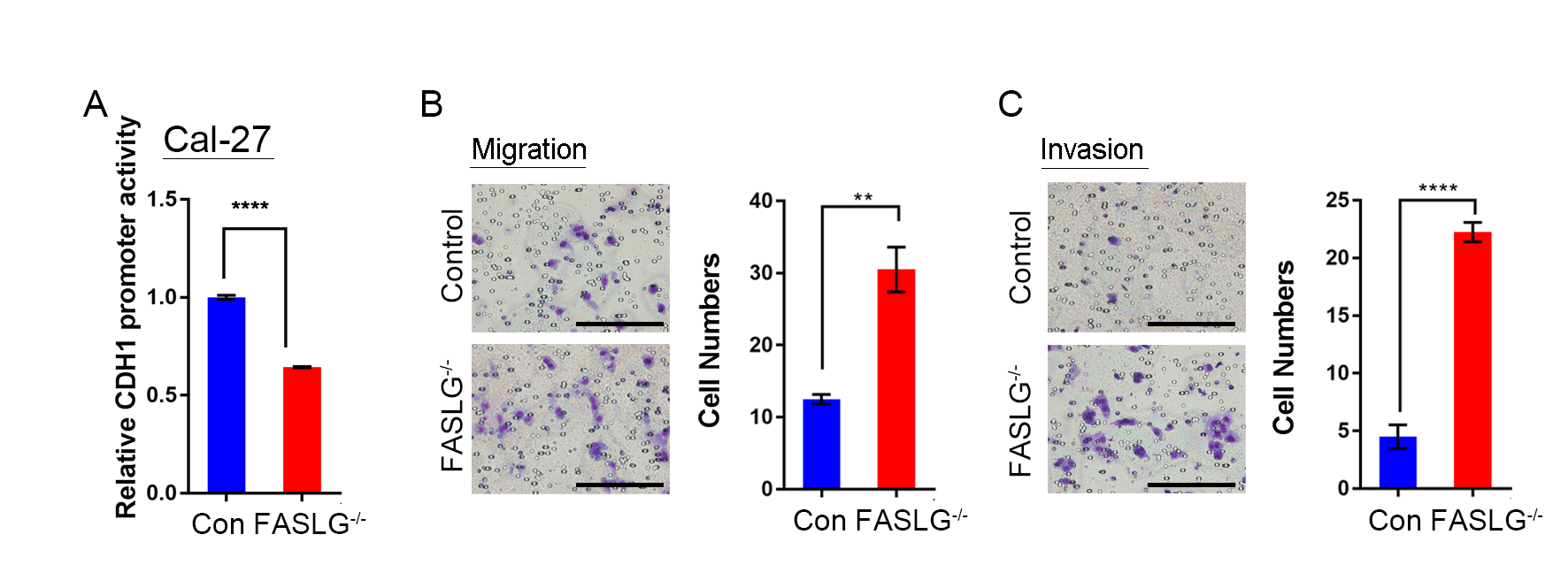

Supplement: Supplementary file 2 — Supplementary Fig. 1. [file 41420_2022_899_MOESM2_ESM.tif]

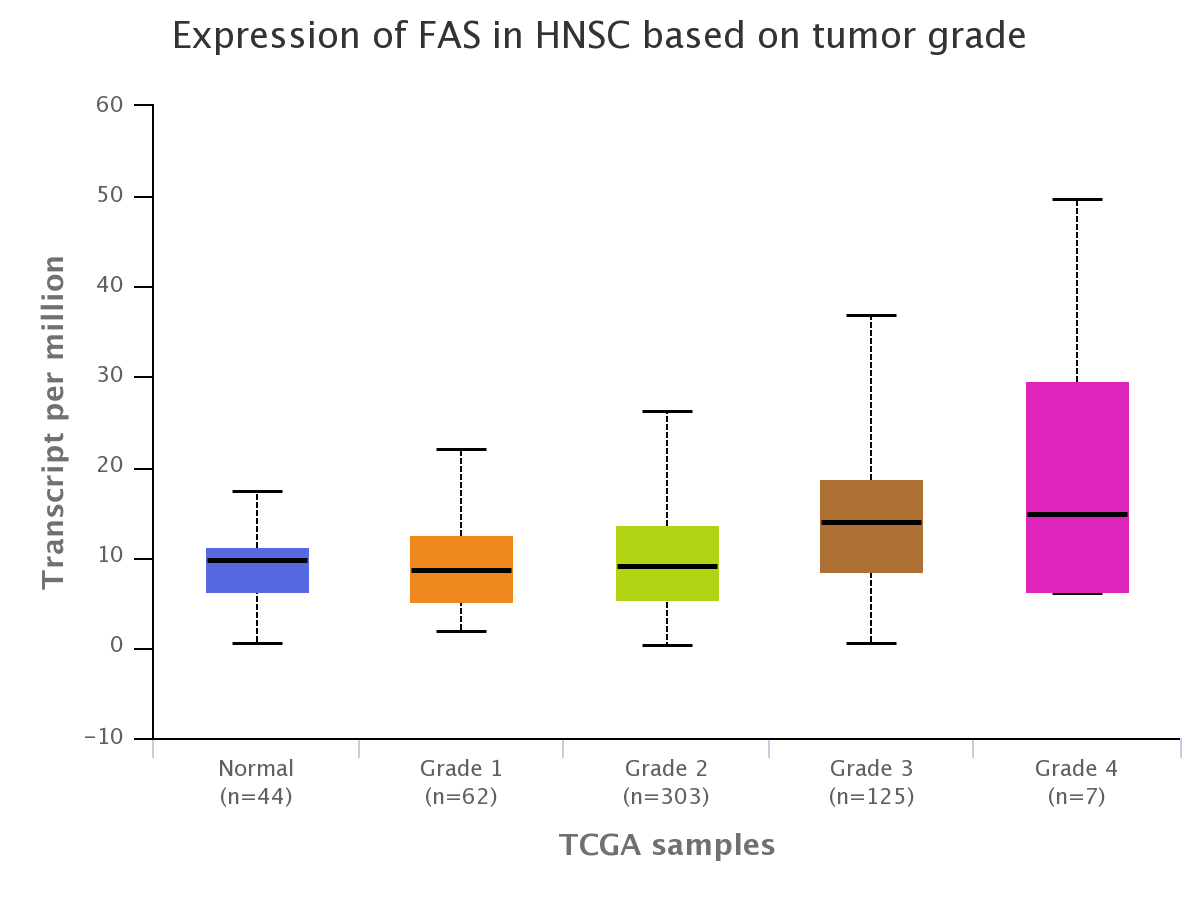

Supplement: Supplementary file 3 — Supplementary Fig. 2. [file 41420_2022_899_MOESM3_ESM.png]

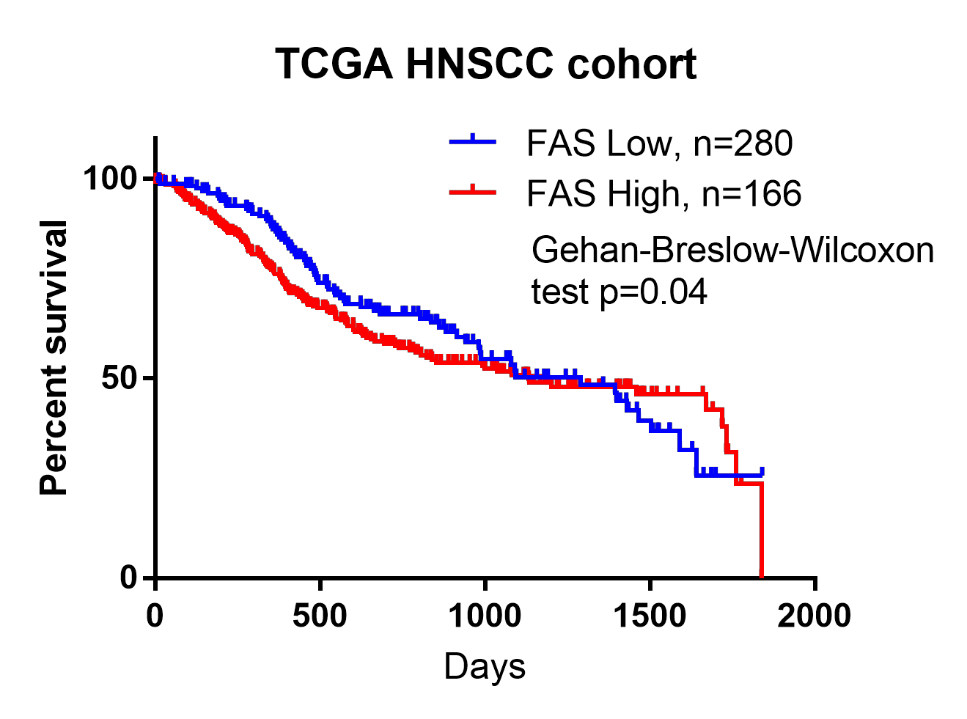

Supplement: Supplementary file 4 — Supplementary Fig. 3. [file 41420_2022_899_MOESM4_ESM.tif]

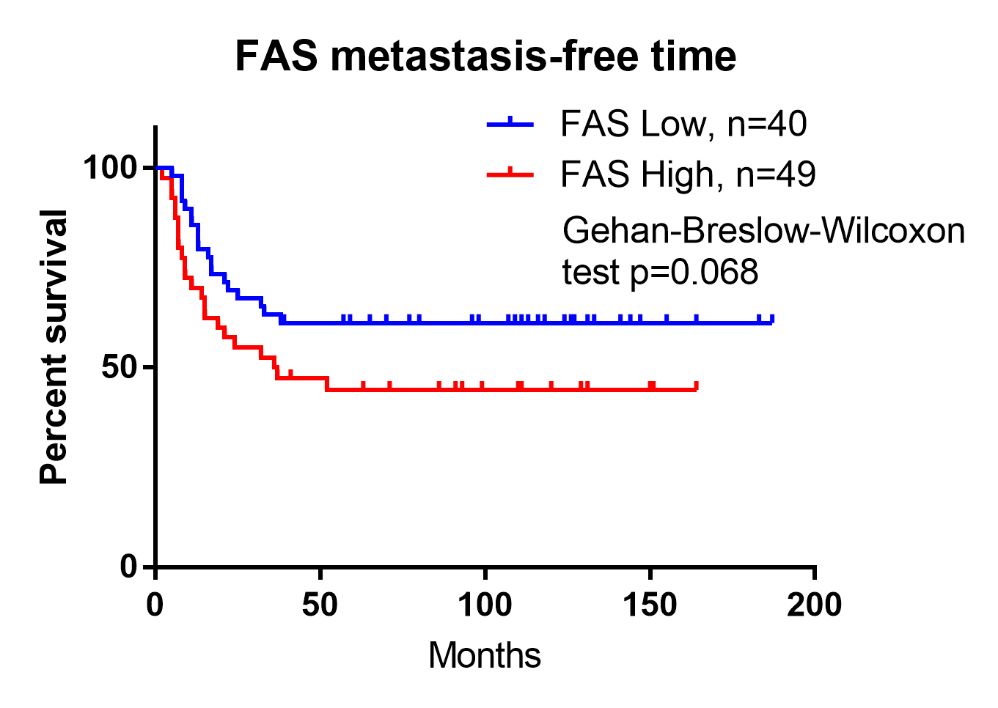

Supplement: Supplementary file 5 — Supplementary Fig. 4. [file 41420_2022_899_MOESM5_ESM.tif]

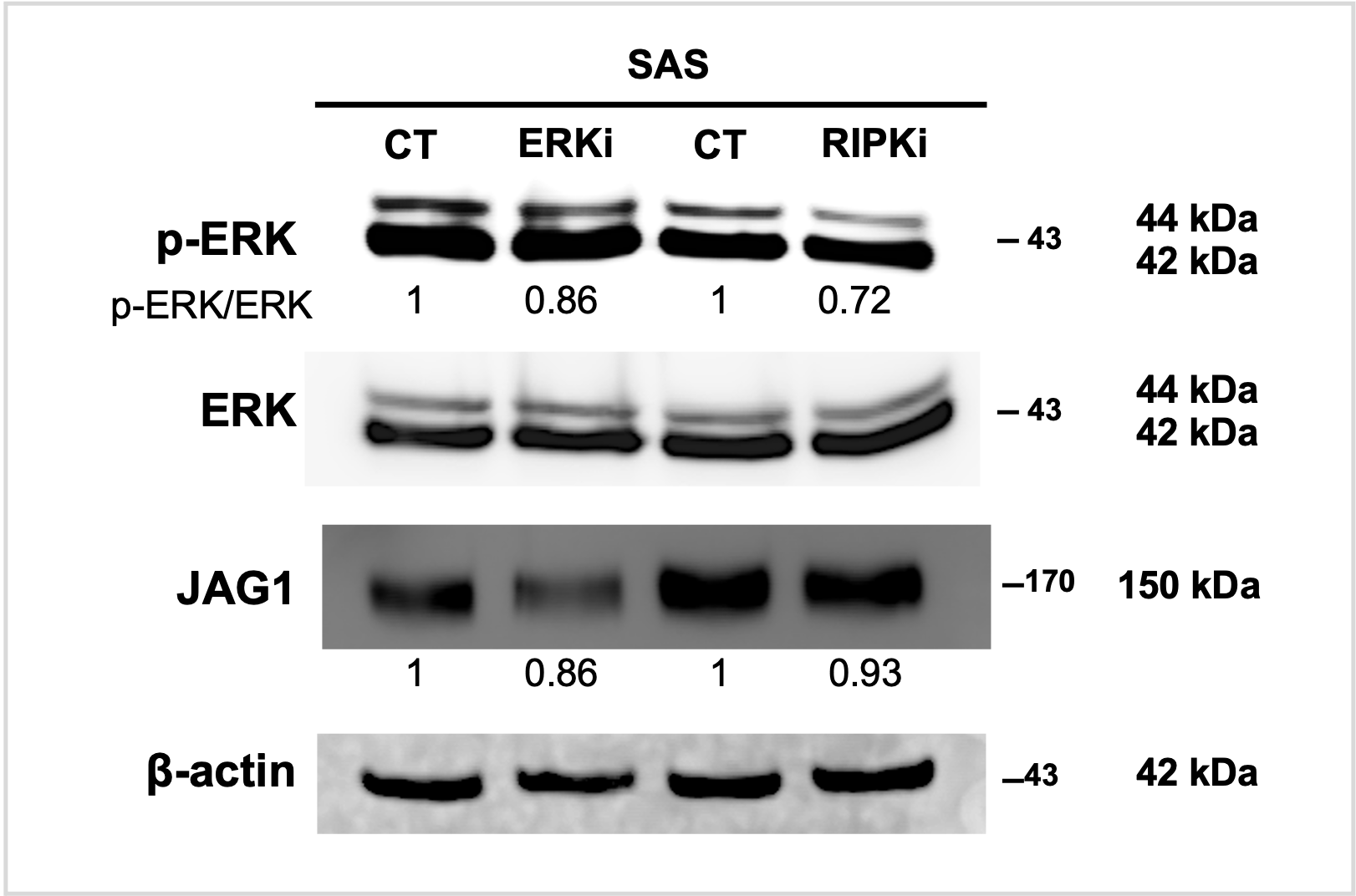

Supplement: Supplementary file 6 — Supplementary Fig. 5. [file 41420_2022_899_MOESM6_ESM.png]

**Figure 1A**


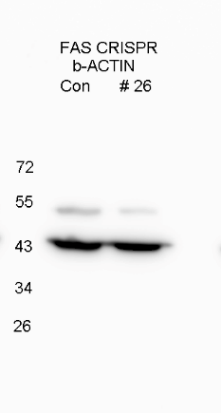

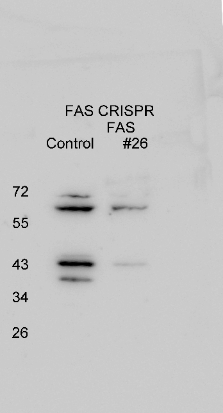


**Figure 4B**

**
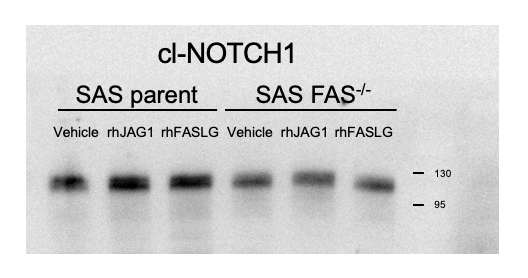

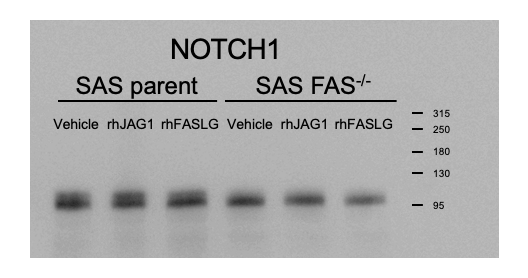

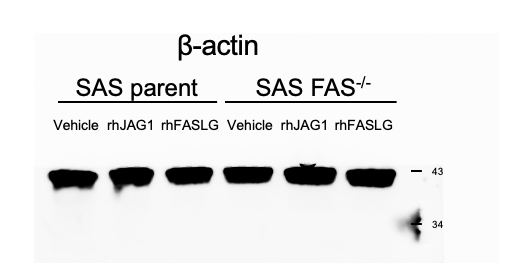
**

**Figure 4C**


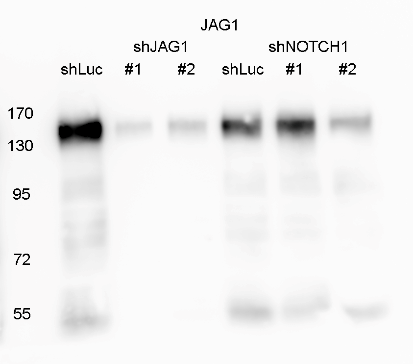

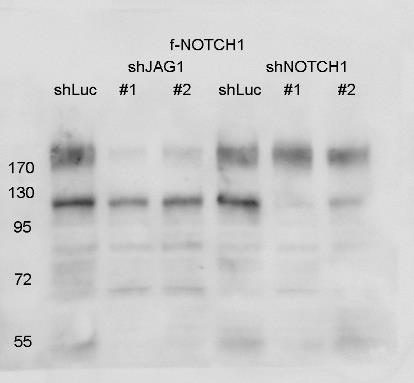

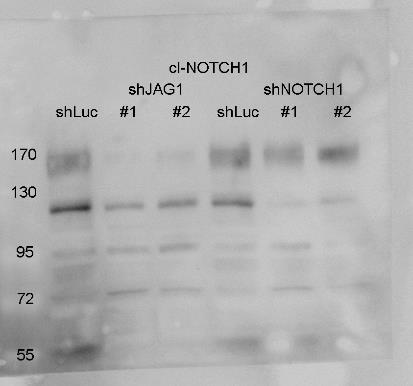

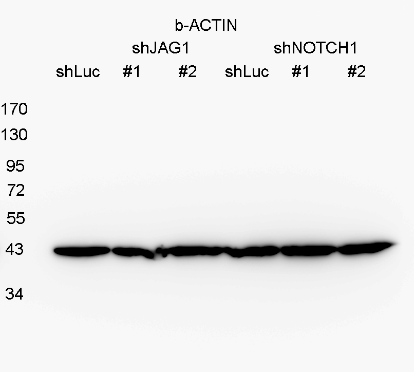


**Figure 7A**


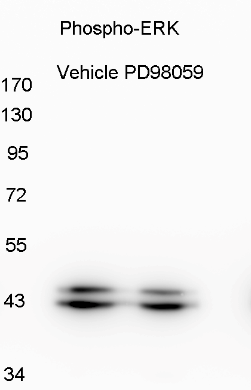

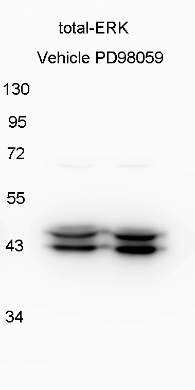


**Figure 7B**


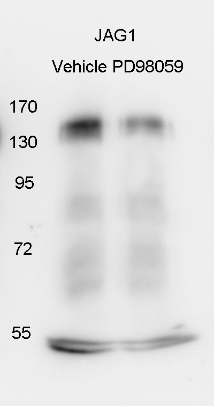

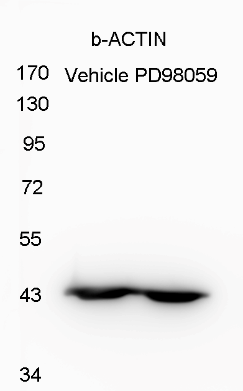


**Figure 7C**


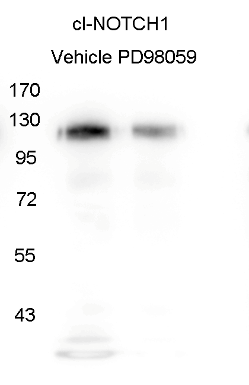

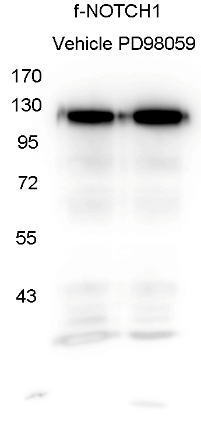


**Supplementary Fig. 5.**


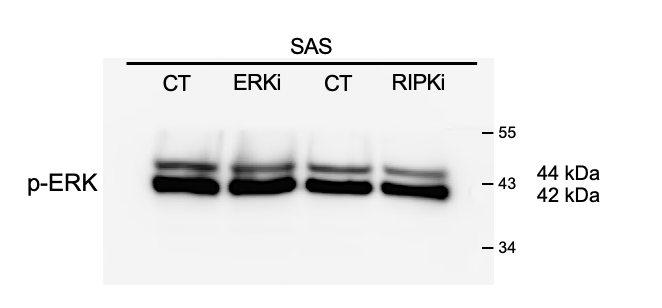


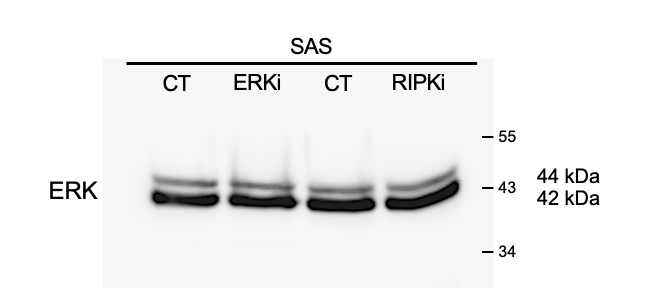


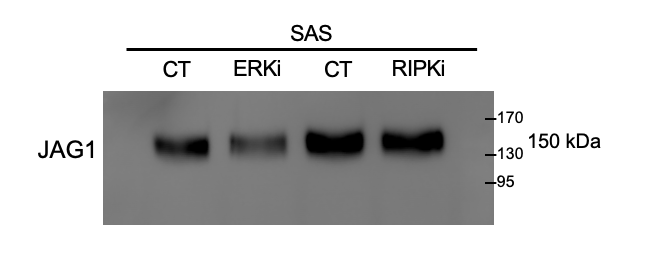


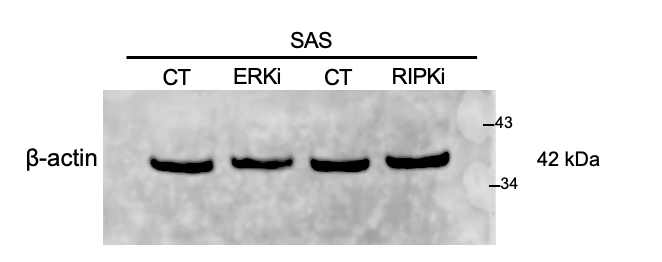

Supplement: Supplementary file 11 — Western blots data [file 41420_2022_899_MOESM11_ESM.docx]
