# Supplementary material for: FAS receptor regulates NOTCH activity through ERK-JAG1 axis activation and controls oral cancer stemness ability and pulmonary metastasis
Source: Cell Death Discov. 2022 Mar 5;8:101. doi: 10.1038/s41420-022-00899-5 (PMC8898312; doi:10.1038/s41420-022-00899-5)
Supplement: Supplementary file 7 — Supplementary Table 1. [file 41420_2022_899_MOESM7_ESM.docx]

**Supplementary Table S1.** **Reagents, vectors, antibody dilution conditions and primer information**

| **Chemical reagents** | | |
| --- | --- | --- |
| **Name** | **Brand** | **Catalogue number** |
| ONE-Glo™ Luciferase Assay System | Promega | E6110 |
| HE Swift Cloning Kit | Biotools Co. | TB-VTT-BB05 |
| FAS ligand recombinant protein | R & D | 126-FL-010 |
| JAG1 recombinant protein | Sinobiological | 11648-H08H |
| PD98059 | Sigma-Al | P215 |
| OmicsGreen qPCR MasterMix. | Omics Bio | QE3933 |
| FBS | Invitrogen | 16000044 |
| DMEM/F12 | Invitrogen | 12500096 |
| DMEM | Invitrogen | 12100046 |
| PSG | Invitrogen | 10378016 |
| bFGF | Invitrogen | 13256029 |
| B27 | Invitrogen | 17504044 |
| EGF | Invitrogen | PHG0311 |
| Human Phospho-Kinase Array | R&D | ARY003B |
| Lipofetamine 2000 | Invitrogen | 11668027 |
| Opti-MEM Media | Invitrogen | 31985062 |
| ERK inhibitor (ERKi; FR-180204) | Selleck | S7524 |
| RIP kinase inhibitor (RIPKi; Necrostatin-1) | Selleck | S8037 |
| **Vectors** | | |
| **Name** | **Brand** | **Catalogue number** |
| pAll-Cas9.Ppuro | RNAiCore | C6-8-67 |
| pLKO.1-shLuc | RNAiCore | - |
| pLKO-shJAG1-1 | RNAiCore | TRCN0000244410 |
| pLKO-shJAG1-2 | RNAiCore | TRCN0000244205 |
| pLKO-shNOTCH1-1 | RNAiCore | TRCN0000003358 |
| pLKO-shNOTCH1-2 | RNAiCore | TRCN0000003360 |
| 7TFP CDH1 reporter | Addgene | 91704 |
| Notch1-ICD-pcw107-V5 | Addgene | 64622 |
| Luciferase-pcw107-V5 | Addgene | 64649 |
| pGreenFire1-Oct4 (EF1α-puro) Lentivector | SBI | TR039PA-P |
| pGL4.26[luc2/minP/Hygro] | Promega | E8441 |
| pGL4[Luc2P/p53-RE/Puro] | Promega | E3651 |
| pGL4.43[luc2P/XRE/Hygro] | Promega | E4121 |
| pGL4[Luc2P/TCF-LEF-RE/Hygro] | Promega | E4611 |
| pGL4[Luc2P/hID1/Hygro] | Promega | CS177202 |
| pGL4.48[luc2p/SBE/Hygro] | Promega | E3671 |
| pGL4[Luc2P/hIL-2/Puro] | Promega | E6761 |
| pGL4.34[luc2P/SRF-RE/Hygro] | Promega | E1350 |
| pGL4.33[luc2P/SRE/Hygro] | Promega | E1340 |
| pGL4.37[luc2P/ARE/Hygro] | Promega | E3641 |
| pGL4[Luc2P/RBP-JK-RE/Hygro] | Promega | CS173601 |
| pGL4[Luc2P/Myc/Hygro] | Promega | CS180201 |
| pGL4[Luc2P/hIL-8/Hygro] | Promega | E8481 |
| pGL4[Luc2P/LCN2/Hygro] | Promega | CS188002 |
| pGL4.42[luc2P/HRE/Hygro] | Promega | E4001 |
| pGL4[Luc2P/Gli-RE/Hygro] | Promega | CS171301 |
| pGL4.45[luc2P/ISRE/Hygro] | Promega | E4141 |
| pGL4[Luc2P/GAS-RE/Hygro] | Promega | CS179301 |
| pGL4.47[luc2P/SIE/Hygro] | Promega | E4041 |
| pGL4[Luc2P/STAT4-RE/Hygro] | Promega | CS181501 |
| pGL4.52[luc2P/STAT5/Hygro] | Promega | E4651 |
| pGL4.32[luc2P/NF-κB-RE/Hygro] | Promega | E8491 |
| pGL4.29[luc2P/CRE/Hygro] | Promega | E8471 |
| pGL4.30[luc2P/NFAT-RE/Hygro] | Promega | E8481 |

| **Antibodies** | | | |
| --- | --- | --- | --- |
| **Name** | **Brand** | **Catalogue number** | **Dilution** |
| β-Actin | Sigma | HPA041271 | 1:5000 |
| FAS | Proteintech | 13098-1-AP | 1:1000 (WB), IHC (1:50) |
| FAS-NEUT (ZB4) | Sigma | 05-338 | 5 μg/ml |
| Phosphor-ERK | Cell Signaling | 9101S | 1:2000 |
| ERK | Cell Signaling | 4696S | 1:2000 |
| JAG1 | Cell Signaling | 70109 | 1:2000 |
| Cleavage Notch1 | Cell Signaling | 4147S | 1:2000 |
| Total Notch1 | Cell Signaling | 3608S | 1:2000 |

| **Primer sequence** | |
| --- | --- |
| **Primer** | **Sequence (5’— 3’)** |
| FAS-CRISPR-F | ACT TGG AAG GCC TGC ATC AT |
| FAS-CRSIPR-R | CTT TCA CTG TAA TCT CTG GA |
| JAG1-F120-139 | GTC CAT GCA GAA CGT GAA CG |
| JAG1-R255-236 | GCG GGA CTG ATA CTC CTT GA |
| GAPDH-F | GAA GGT GAA GGT CGG AGT |
| GAPDH-R | GAA GAT GGT GAT GGG ATT TC |
