# Supplementary material for: FAS receptor regulates NOTCH activity through ERK-JAG1 axis activation and controls oral cancer stemness ability and pulmonary metastasis
Source: Cell Death Discov. 2022 Mar 5;8:101. doi: 10.1038/s41420-022-00899-5 (PMC8898312; doi:10.1038/s41420-022-00899-5)
Supplement: Supplementary file 8 — Supplementary Table 2. [file 41420_2022_899_MOESM8_ESM.docx]

**Supplementary Table S2. Clinical characteristics of FAS in validation HNSCC cohort**

| **FAS** | | **FAS Expression** | |  | |
| --- | --- | --- | --- | --- | --- |
|  |  | **High** | **Low** | |  |
|  |  | **(n =58)** | **(n=59)** | | ***p* value** |
| **Age (Years)** |  | 57.02±14.16 | 54.40±13.78 | | 0.32 |
| **T status** | T1+T2 | 35 (29.91%) | 46 (39.32%) | | 0.039* |
|  | T3+T4 | 23 (19.66%) | 13 (11.11%) | |  |
| **N status** | N0 | 34 (29.06%) | 46 (39.32%) | | 0.024* |
|  | N1-3 | 24 (20.51%) | 13 (11.11%) | |  |
| **Stage** | I+II | 23 (20.00%) | 38 (33.04%) | | 0.591 |
|  | III+IV | 33 (28.70%) | 21 (18.26%) | |  |
| **Differentiation** | WD | 11 (9.40%) | 24 (20.51%) | | 0.010* |
|  | MD+PD | 47 (40.17%) | 35 (29.91%) | |  |
| **Perineural invasion** | No | 26 (22.61%) | 28 (24.35%) | | 0.912 |
|  | Yes | 30 (26.09%) | 31 (26.96%) | |  |
| **Angiolymphatic invasion** | No | 38 (33.04%) | 36 (31.30%) | | 0.444 |
|  | Yes | 18 (15.65%) | 23 (20.00%) | |  |

WD: well differentiation; MD: mild differentiation; PD: poor differentiation.

* Indicated *p* < 0.05
