# Supplementary material for: FAS receptor regulates NOTCH activity through ERK-JAG1 axis activation and controls oral cancer stemness ability and pulmonary metastasis
Source: Cell Death Discov. 2022 Mar 5;8:101. doi: 10.1038/s41420-022-00899-5 (PMC8898312; doi:10.1038/s41420-022-00899-5)
Supplement: Supplementary file 12 — Attribution form [file 41420_2022_899_MOESM12_ESM.docx]

**FAS Receptor Regulates NOTCH Activity through ERK-JAG1 Axis Activation and Controls Oral Cancer Stemness Ability and Pulmonary Metastasis**

Li-Jie Li^1,2^, Peter Mu-Hsin Chang^3,4,5^, Chien-Hsiu Li^2^, Yu-Chan Chang^6^, Tsung-Ching Lai^2,7^, Chia-Yi Su^2,8^, Chi-Long Chen^9,10^, Wei-Min Chang^2, 11#^, Michael Hsiao ^2,12,13#^, Sheng-Wei Feng^14,15#^

**Author Contributions Statement**

**Conception and design:** L. J. Li, P.M.H. Chang, W.M. Chang, and M. Hsiao and S.W. Feng

**Development of methodology:** L.J Li, Y.C. Chang, T.C. Lai, W.M. Chang, and M. Hsiao

**Acquisition of data (provided animals, acquired and managed patients, provided facilities, etc.)**: C. L. Chen and M. Hsiao

**Analysis and interpretation of data (e.g., statistical analysis, biostatistics, computational analysis):** L. J. Li, P.M.H. Chang, C. H. Li, Y. C. Chang, T. C. Lai, and C. Y. Su

**Writing, review, and/or revision of the manuscript:** L. J. Li, P.M.H. Chang, C. H. Li, Y. C. Chang, T. C. Lai, and C. Y. Su, W.M. Chang, and M. Hsiao and S.W. Feng

**Administrative, technical, or material support (i.e., reporting or organizing data, constructing databases):** P.M.H. Chang, Y. C. Chang, W.M. Chang, and M. Hsiao and S.W. Feng

**Study supervision:** W.M. Chang, and M. Hsiao and S.W. Feng
