# Supplementary material for: FAS receptor regulates NOTCH activity through ERK-JAG1 axis activation and controls oral cancer stemness ability and pulmonary metastasis
Source: Cell Death Discov. 2022 Mar 5;8:101. doi: 10.1038/s41420-022-00899-5 (PMC8898312; doi:10.1038/s41420-022-00899-5)
Supplement: Supplementary file 13 — Authorship change form [file 41420_2022_899_MOESM13_ESM.pdf]

SPRINGER NATURE Change of authorship request form - Journals (pre-acceptance)

Important information. Please read.

- This form should be used by authors to request any change in authorship (adding/deleting authors) including changes in corresponding authors. This form should not be used for name changes. Please fully complete all sections. Use black ink and block capitals and provide each author's full name with the given name first followed by the family name.

- By signing this declaration, all authors guarantee that the order of the authors are in accordance with their scientific contribution, if applicable as different conventions apply per discipline, and that only authors have been added who made a meaningful contribution to the work.

- Please note, in author collaborations where there is formal agreement for representing the collaboration, it is sufficient for the representative or legal guarantor (usually the corresponding author) to complete and sign the Authorship Change Form on behalf of all authors, **next to the added/removed author(s)**. **(Complete Section 3, followed by Section 6.)**
  In author collaborations where there is no formal agreement for representing the collaboration and **there are more than 10 authors**, one may sign for all, provided the signer appends correspondence that attests that each of the authors have agreed to the change **and the added/removed authors sign the form**. **(Complete Section 3, followed by Section 6.)**

- Please note, we cannot investigate or mediate any authorship disputes. If you are unable to obtain agreement from all authors (including those who you wish to be removed) you must refer the matter to your institution(s) for investigation. Please inform us if you need to do this.

- If you are not able to return a fully completed form within **30 days** of the date that it was sent to the author requesting the change, we may have to withdraw your manuscript. We cannot publish manuscripts where authorship has not been agreed by all authors (including those who have been removed).

- Incomplete forms will be rejected.

- Please return/upload this form, fully completed, to the Journals Editorial Office. The Journal and/or Publisher will consider the information you have provided to decide whether to approve the proposed change in authorship. We may decide to contact your institution for more information or undertake a further investigation, if appropriate, before making a final decision.

SPRINGER NATURE

Change of authorship request form - Journals (pre-acceptance)

Section 1: Please provide the current title of manuscript

Manuscript ID no.: CDDISCOVERY-21-1958R2

Title: FAS Receptor Regulates NOTCH Activity through ERK-JAG1 Axis Activation and Controls Oral Cancer Stemness Ability and Pulmonary Metastasis

Section 2: Please provide the previous authorship, in the order shown on the manuscript before the changes were introduced. Please indicate the corresponding author by adding (CA) behind the name.

	First name(s)	Family name	ORCID or SCOPUS id, if available
1st author	Wei-Min	Chang	
2nd author	Li-Jie	Li	
3rd author	Chien-Hsiu	Li	0000-0002-4648-3744
4th author	Yu-Chan	Chang	
5th author	Tsung-Ching	Lai	
6th author	Chia-Yi	Su	
7th author	Chi-Long	Chen	
8th author	Peter Mu-Hsin (CA)		
9th author	Michael (CA)	Hsiao	0000-0001-8529-9213
10th author			

Please use an additional sheet if there are more than 10 authors.

SPRINGER NATURE Springer Nature is one of the world's leading global research, educational and professional publishers, created in May 2015 through the combination of Nature Publishing Group, Palgrave Macmillan, Macmillan Education and Springer Science+Business Media.

SPRINGER NATURE

Change of authorship request form - Journals (pre-acceptance)

Section 3: Please provide a justification for change. Please use this section to explain your reasons for changing the authorship of your manuscript, e.g. what necessitated the change in authorship? Please refer to the (journal) policy pages for more information about authorship. Please explain why omitted authors were not originally included and/or why authors were removed on the submitted manuscript.

We added Sheng-Wei Feng due to his contribution in revision.

He, as a study supervisor, provided valuable suggestions and assisted in collaboration with authors and answering some queries raised by reviewers.

Section 4: Proposed new authorship. Please provide your new authorship list in the order you would like it to appear on the manuscript. Please indicate the corresponding author by adding (CA) behind the name. <u>If the Corresponding Author has changed, please indicate the reason under section 3</u>.

	First name(s)	Family name (this name will appear in full on the final publication and will be searchable in various abstract and indexing databases)	Affiliated institute	E-mail address
1st author	Li-Jie	Li 1,2	1 Ph.D. Program of School of Dentistry, College of Oral Medicine, Taipei Medical University, Taipei, Taiwan<br>2 Genomics Research Center, Academia Sinica, Taipei, Taiwan	xu3xu4ru56lily@gmail.com
2nd author	Peter Mu-Hsin	Chang 3,4,5	3 Department of Oncology, Taipei Veterans General Hospital, Taipei, Taiwan	i54821413@yahoo.com.tw
3rd author	Chien-Hsiu	Li 2	4 Faculty of Medicine, College of Medicine, National Yang Ming Chiao Tung University, Taipei, Taiwan<br>5 Institute of Biopharmaceutical Sciences, National Yang Ming Chiao Tung University, Taipei, Taiwan	dicknivek@icloud.com
4th author	Yu-Chan	Chang 6	6 Department of Biomedical Imaging and Radiological Sciences, National Yang Ming Chiao Tung University, Taipei, Taiwan	jameskobe0@gmail.com
5th author	Tsung-Ching	Lai 2,7	7 Division of Pulmonary Medicine, Department of Internal Medicine, Wan Fang Hospital, Taipei Medical University, Taipei<br>8 Department of Biomedical Engineering, Johns Hopkins University, Baltimore, MD, USA	chuching0305@gmail.com
6th author	Chia-Yi	Su 2,8	9 Department of Pathology, College of Medicine, Taipei Medical University, Taipei, Taiwan	joysuforlab@gmail.com
7th author	Chi-Long	Chen 9,10	10 Department of Pathology, Taipei Medical University Hospital, Taipei Medical University, Taipei, Taiwan.<br>11 School of Oral Hygiene, College of Oral Medicine, Taipei Medical University, Taipei, Taiwan	chencl@tmu.edu.tw
8th author	Wei-Min (CA)	Chang 2, 11	12 Department of Biochemistry, Kaohsiung Medical University, Kaohsiung, Taiwan	weiminchang@tmu.edu.tw
9th author	Michael (CA)	Hsiao 2,12,13	13 Ph.D. Program of Translational Medicine, Taipei Medical University, Taipei, Taiwan<br>14 School of Dentistry, College of Oral Medicine, Taipei Medical University, Taipei, Taiwan	mhsiao@gate.sinica.edu.tw
10th author	Sheng-Wei (CA)	Feng 14,15	15 Division of Prosthodontics, Department of Dentistry, Taipei Medical University Hospital, Taipei, Taiwan	shengwei@tmu.edu.tw

Please use an additional sheet if there are more than 10 authors.

SPRINGER NATURE Springer Nature is one of the world's leading global research, educational and professional publishers, created in May 2015 through the combination of Nature Publishing Group, Palgrave Macmillan, Macmillan Education and Springer Science+Business Media.

Change of authorship request form - Journals (pre-acceptance)

Section 5: Author contribution, Acknowledgement and Disclosures. Please use this section to provide a new disclosure statement and, if appropriate, acknowledge any contributors who have been removed as authors and ensure you state what contribution any new authors made (if applicable per the journal or book (series) policy). Please ensure these are updated in your manuscript - after approval of the change(s) - as our production department will not transfer the information in this form to your manuscript.

New acknowledgements:

The authors would like to acknowledge the great help and assistance of Experimental Animal Imaging and Molecular Pathology Core Facilities and RNAi Core facility of Genomic Research Center, Academia Sinica (Taipei, Taiwan).

New Disclosures (financial and non-financial interests, funding):

Consent for publication

All authors approved to publish the study in this journal.

Funding

The study was financially supported by the Academia Sinica [AS-SUMMIT-108] to Michael Hsiao and MOST 107-2627-M-075 -001 to Peter Mu-Hsin Chang and finical support by Taipei Medical University [TMU108-AE1-B52] to Wei-Min Chang

New Author Contributions statement (if applicable per the journal policy):

Conception and design: L. J. Li, P.M.H. Chang, W.M. Chang, and M. Hsiao and S.W. Feng
Development of methodology: L.J Li, Y.C. Chang, T.C. Lai, W.M. Chang, and M. Hsiao
Acquisition of data (provided animals, acquired and managed patients, provided facilities, etc.): C. L. Chen and M. Hsiao
Analysis and interpretation of data (e.g., statistical analysis, biostatistics, computational analysis): L. J. Li, P.M.H. Chang, C. H. Li, Y. C. Chang, T. C. Lai, and C. Y. Su
Writing, review, and/or revision of the manuscript: L. J. Li, P.M.H. Chang, C. H. Li, Y. C. Chang, T. C. Lai, and C. Y. Su, W.M. Chang, and M. Hsiao and S.W. Feng
Administrative, technical, or material support (i.e., reporting or organizing data, constructing databases): P.M.H. Chang, Y. C. Chang, W.M. Chang, and M. Hsiao and S.W. Feng
Study supervision: W.M. Chang, and M. Hsiao and S.W. Feng

State 'Not applicable' if there are no new authors.

SPRINGER NATURE Springer Nature is one of the world's leading global research, educational and professional publishers, created in May 2015 through the combination of Nature Publishing Group, Palgrave Macmillan, Macmillan Education and Springer Science+Business Media.

SPRINGER NATURE

Change of authorship request form - Journals (pre-acceptance)

Section 6: Declaration of agreement. *All* authors, unchanged, new and removed *must* sign this declaration.

(NB: Please print the form, (docu)-sign and return/upload a scanned copy. Please note that signatures that have been inserted as an image file are acceptable as long as it is handwritten. Typed names in the signature box are unacceptable.) *** Please delete as appropriate. Delete all of the bold if you were on the original authorship list and are remaining as an author.**

	First name	Family name		Signature	Date
1st author	Li-Jie	Li	I agree to the proposed new authorship shown in section 4 **/and the addition/removal*of my name to the authorship list** /and the proposed change in corresponding author	LI, LI-JIE	2022.02.09
2nd author	Peter Mu-Hsin	Chang	I agree to the proposed new authorship shown in section 4 **/and the addition/removal*of my name to the authorship list** /and the proposed change in corresponding author	[illegible]	2022.02.10
3rd author	Chien-Hsiu	Li	I agree to the proposed new authorship shown in section 4 **/and the addition/removal*of my name to the authorship list** /and the proposed change in corresponding author	Chien-Hsiu Li	2022.02.10
4th authors	Yu-Chan	Chang	I agree to the proposed new authorship shown in section 4 **/and the addition/removal*of my name to the authorship list** /and the proposed change in corresponding author	Yu-Chan, Chang	2/9/2022
5th author	Tsung-Ching	Lai	I agree to the proposed new authorship shown in section 4 **/and the addition/removal*of my name to the authorship list** /and the proposed change in corresponding author	Lai, Tsung-Ching	2022. 2. 10
6th author	Chia-Yi	Su	I agree to the proposed new authorship shown in section 4 **/and the addition/removal*of my name to the authorship list** /and the proposed change in corresponding author	Chia-Yi Su	Feb 09, 2022
7th author	Chi-Long	Chen	I agree to the proposed new authorship shown in section 4 **/and the addition/removal*of my name to the authorship list** /and the proposed change in corresponding author	Chi-Long Chen	2022.02.10

SPRINGER NATURE Springer Nature is one of the world's leading global research, educational and professional publishers, created in May 2015 through the combination of Nature Publishing Group, Palgrave Macmillan, Macmillan Education and Springer Science+Business Media.

SPRINGER NATURE

Change of authorship request form - Journals (pre-acceptance)

	First name	Family name		Signature	Date
8th author	Wei-Min	Chang	I agree to the proposed new authorship shown in section 4 /**and the addition/removal*of my name to the authorship list** /and the proposed change in corresponding author	Wei-Min Chang	2.10.2022
9th author	Michael	Hsiao	I agree to the proposed new authorship shown in section 4 /**and the addition/removal*of my name to the authorship list** /and the proposed change in corresponding author	[illegible] Michael Hsiao	2/10/2022
10th author	Sheng-Wei	Feng	I agree to the proposed new authorship shown in section 4 /**and the addition/removal*of my name to the authorship list** /and the proposed change in corresponding author	Sheng-Wei Feng	2022.02.10

Please use an additional sheet if there are more than 10 authors.

In case of author collaborations with formal agreement:

	Name of consortium/consortia	First name	Family name		Signature	Date
Representative/legal guarantor				I agree to the proposed new authorship shown in section 4 /**and the addition/removal*of my name to the authorship list** /and the proposed change in corresponding author		

Both added/removed authors should complete the information in the first table under Section 6.

---- End of form ----

SPRINGER NATURE Springer Nature is one of the world's leading global research, educational and professional publishers, created in May 2015 through the combination of Nature Publishing Group, Palgrave Macmillan, Macmillan Education and Springer Science+Business Media.
